# Description of a case of late-onset cryopyrin-associated periodic syndrome due to low-level somatic *NLRP3* mosaicism

**DOI:** 10.1186/1546-0096-13-S1-P60

**Published:** 2015-09-28

**Authors:** A Mensa-Vilaro, MT Bosque-Peralta, E Gonzalez-Roca, M Casorran-Berges, C Delgado-Beltran, S Plaza, MC Anton, E Ruiz-Ortiz, J Yagüe, JI Arostegui

**Affiliations:** 1 Hospital Clínic, Immunology, Barcelona, Spain; 2 Hospital Clinico Universitario Lozano Blesa, Zaragoza, Spain

## Introduction

Cryopyrin-associated periodic syndromes (CAPS) usually present in early childhood as an urticaria-like skin rash associated with an increased inflammatory response, with additional manifestations (i.e. arthropathy, AA amyloidosis or deafness) typically restricted to certain phenotypes. CAPS are caused by dominantly inherited or *de novogain-of-functionNLRP3* mutations. The introduction of next-generation sequencing (NGS) into clinics has revealed the important role of somatic *NLRP3* mosaicism in these syndromes by means of its detection in a high proportion of patients who were apparently mutation-negative by Sanger sequencing. Thus, NGS technologies are becoming essential for routinely identifying the genetic cause of the suspected autoinflammatory disease.

## Objective

To describe a Spanish patient with CAPS that start in adulthood in whom molecular analyses detected a novel *NLRP3* mutation as a somatic mutation.

## Patients and methods

Genomic DNA was extracted from peripheral blood. The analysis of the six most common genes associated with autoinflammatory diseases (*MEFV*, *TNFRSF1A*, *MVK*, *NLRP3*, *NOD2* and *PSTPIP1*) was performed by NGS. Additional molecular studies of somatic *NLRP3* mosaicism were performed by targeted deep sequencing (TDS).

## Results

The patient is a 63 year-old Spanish male who presented with a generalized urticarial rash, a gradually worsening oligoarthritis at wrists, elbows and knees, and a moderate bilateral sensorineural hypoacusia starting in his 50s. Laboratory results showed a marked leucocytosis, neutrophilia and increased inflammatory markers without evidence of circulating autoantibodies. Multiple therapeutic approaches including NSAID, corticosteroids, methotrexate and colchicine result in poor or partial responses. Screening of autoinflammatory-associated genes identified a novel *NLRP3* variant (c.1906C>G; p.Gln636Glu) with an allele frequency of 12.2% (coverage: 738x). We hypothesized that this variant could be a somatic *NLRP3* mutation. TDS confirmed the somatic *NLRP3* mosaicism at 18.4 % (mean of triplicates; mean coverage: 6225x). Additional analyses showed that this variant has never been reported in public databases, that is located on a highly evolutionary conserved amino acid residue and that is predicted to be possibly damaging by PolyPhen-2 algorithm. Additional functional and genetic studies are currently ongoing.

## Conclusions

We herein describe the case of a patient with a clinical picture compatible with MWS, with the exception of a late onset of the disease, who carries a somatic *NLRP3* mosaicism. Our findings highlight the diagnostic utility of NGS technologies in detecting low-level somatic gene mosaicism and support its use as a routine genetic screening tool.

## Consent to publish

Written informed consent for publication of their clinical details was obtained from the patient/parent/guardian/relative of the patient.

